# IMRT, RapidArc® and conformal radiotherapy in the treatment of tumours of the anal canal

**DOI:** 10.3332/ecancer.2014.469

**Published:** 2014-10-06

**Authors:** Ricardo Cendales, Jaider Vásquez, Juan Arbelaez, Ivan Bobadilla, Felipe Torres, Armando Gaitan

**Affiliations:** Centro de Control de Cáncer, Bogotá 110221, Colombia

**Keywords:** anus neoplasms, conformal radiotherapy, intensity-modulated radiotherapy

## Abstract

**Purpose:**

To compare dosimetric results of the use of RapidArc® with simultaneous integrated boost, sliding window intensity-modulated radiotherapy (IMRT) with simultaneous integrated boost, and conformal radiotherapy with sequential boost in the management of anal canal cancer.

**Methods:**

Two patients with squamous cell cancer of the anal canal with compromised inguinal nodes were included. The simulation was performed in the supine position with a customized Vac-Lok™ immobilizer. Treatment volumes and organs at risk were defined in accordance with international recommendations. Dosimetric comparisons were made in the target volume by means of tumour conformity, coverage, and homogeneity indexes; in healthy organs, integral doses were compared.

**Results:**

A similar planning target volume coverage was achieved with the three techniques. The two IMRT techniques demonstrated benefits in doses received by healthy organs compared to the conformal radiotherapy. RapidArc® showed reduction in the execution time and monitor units required for treatment compared with sliding window IMRT.

**Conclusions:**

The IMRT showed coverage and tumour conformity indexes similar to those of conformal radiotherapy with better dosimetric results in the organs at risk, which should translate into a better toxicity profile. RapidArc® demonstrated benefits over the sliding window IMRT, which makes treatment more comfortable for the patient with less uncertainty about intrafraction motion and a reduced potential for radiation-induced tumours.

## Introduction

Colon, rectal and anal cancer is the third cause of cancer incidence and mortality in Colombia [1, 2]. The current standard in the management of squamous cell cancer of the anal canal is based on radiotherapy in association with chemotherapy with 5-Fluorouracil and Mitomycin-C [3]. Traditional fields of radiation treatment performed by conventional technique are broad since they include iliac and inguinal nodes and require that the skin of the perianal region be included with a margin of safety. Consequently, various structures, such as the intestine, bladder, iliac wings, genitals and genital and inguinal skin end up receiving radiation dose, that coupled with chemotherapy; determine important toxicity, which is usually the cause of interruptions, abandonment of treatment, and even death due to treatment toxicity [4].

The radiation treatment can be done by means of various techniques, such as conventional, conformal, intensity-modulated radiotherapy (IMRT) with direct modulation, IMRT with inverse planning or IMRT with dynamic arc (RapidArc®). Several dosimetric studies have shown the benefits of IMRT with inverse planning in patients with anal canal cancer since a better dose homogeneity is achieved on the target volume while the doses received by the genitals, bladder, intestine, genital and inguinal skin, iliac wings and femoral heads are decreased [5]. RapidArc® techniques also allow for the decrease of monitor units and the daily execution time of treatment compared to standard IMRT techniques [6].

The objective of this study is to compare, in the local context, the dosimetric results with the use of RapidArc® with simultaneous integrated boost, sliding window IMRT with simultaneous integrated boost, and conformal radiotherapy with sequential boost in the treatment of two typical patients with locally advanced anal canal cancer. This comparison will be set in terms of coverage, homogeneity, tumour conformity, monitor units, execution times, and dose indexes received by healthy tissues, such as the genitals, bladder, skin of the inguinal region, intestine, femoral heads, and iliac wings.

## Patients and methods

### Patients

Two patients (one male and one female) with T3 anal canal cancer and compromised inguinal nodes were selected.

### Simulation

Both patients were simulated in the supine position, with legs spread in the commonly known ‘frog’ position with a customized vacuum Vac-Lok™ immobilization device. A three-dimensional simulation with 3-mm-thick axial sections was performed on dedicated computed tomography (CT) scanning equipment.

### Treatment volumes

A radiation oncologist with expertise in IMRT delimited the gross tumour volume (GTV) based on the findings of the simulation CT scan, the physical examination, the diagnostic CT and MRI and the transrectal ultrasound. The macroscopic disease of both the primary tumour as well as the compromised nodes was included in the GTV. The clinical target value (CTV) 59.4 was defined by adding a 1 cm margin to the GTV, excluding bone structures. The planning target volume (PTV) 59.4 was defined by adding 0.5 cm to the CTV 59.4. The CTV 49.5 was defined as the areas with high risk of compromised tumour growth but that proved to be negative in the previously described studies; included in these areas were the rectal wall, ischioanal fossa, mesorectum, anal canal, perirectal nodes, internal and external iliac nodes, and obturator and inguinal nodes. The PTV 49.5 was defined by adding 1 cm to the CTV 49.5. The definitions of GTV, CTV, and PTV correspond to those recommended on an international level [7, 8].

### Organs at risk

The organs such as the external genitalia—including the penis and testicles for males and the labia majora, labia minora, clitoris, and distal third of the vagina for females—were defined as organs at risk (OARs). Additionally, the iliac wings were demarcated from the level of the acetabula, bladder, intestine, and femoral heads. The healthy tissue was defined by the difference between the contour of the patient and the volume of the PTV.

### Prescription of doses and treatment techniques

Three treatment techniques were compared: conformal radiotherapy with sequential boost; sliding window IMRT with inverse planning and simultaneous integrated boost; IMRT with dynamic arc [volumetric modulated arcotherapy (VMAT)] by means of Varian’s RapidArc® commercial solution—with inverse planning and simultaneous integrated boost.

For the two IMRT plans, a dose of 49.5 Gy in 33 fractions of 1.5 Gy daily was prescribed on the PTV 49.5. For the PTV 59.4, a dose of 59.4 Gy in 33 fractions of 1.8 Gy daily was prescribed. It was established that 95% of the volume of the PTV59.4 should receive 100% of the prescribed dose (V59.4 Gy >= 95%) and that the volume of the PTV that receives 107% or more of the prescribed dose should be less than 2% (V63.5 Gy <= 2%). The other dosimetric objectives are described in Table 1. Concerning the sliding window IMRT technique, nine coplanar fields were employed, whereas for the RapidArc® technique, two arcs were employed.

For the conformal radiotherapy treatment, a first stage was executed up to 45 Gy, in which the PTV 45 was treated in 25 fractions of 1.8 Gy through five pelvic fields. After completing 45 Gy on this PTV, a conformal sequential reinforcement was planned with photons up to 59.4 Gy in the same fractionation on the PTV 59.4.

Eclipse version 8.9 software was employed for the planning. The treatment was administered with a Varian Clinac IX model linear accelerator, equipped with conebeam computerized tomography (CBCT), an electronic portal imaging device (EPID), and Millennium (MMLC-120) 120 multileaf equipment.

### Evaluation of plans

The PTV volume receiving 90% of the prescribed dose (V90), the V95, and V107 were described; and also, the dose that 2% of the volume receives (D2), D50, D95, and D98 were described. The coverage index was calculated as the minimum dose (D98) divided by the prescribed dose, the homogeneity index as the maximum dose (D2) divided by the prescribed dose, and the tumour conformity index as the PTV volume covered by the prescription isodose divided by the total PTV volume. The dosimetric objectives were described for each of the organs at risk were described. The integral dose [9] was calculated as the average dose in Gy multiplied by the volume of the organ measured in litres [10].

## Results

The PTV coverage achieved by the three techniques was similar, whereas the homogeneity was slightly better for the IMRT with dynamic arc. The tumour conformity index was slightly better for the two IMRT techniques.

The execution time of the RapidArc® treatment was 23% longer compared with the time required for conformal radiotherapy. Likewise, the treatment time of sliding window IMRT was 4.9 times longer than that of the conformal radiotherapy.

With regard to monitor units, RapidArc® required an average of 770 UM, which is 3.5 times greater than those required by conformal radiotherapy. The sliding window IMRT required 3288 UM on average, which is 15 times greater than those required by conformal radiotherapy (Table 2).

**Figure 1.**
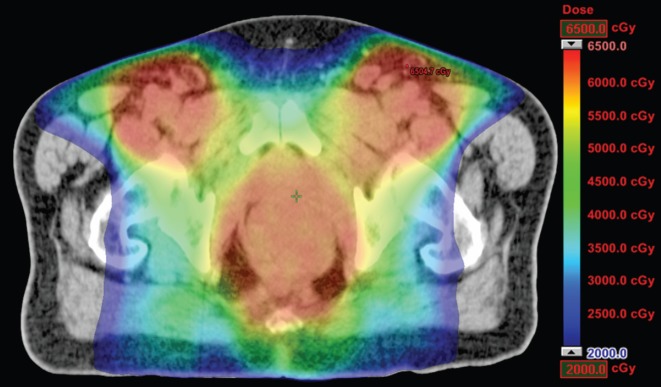
Dosimetric results obtained with RapidArc.

**Figure 2.**
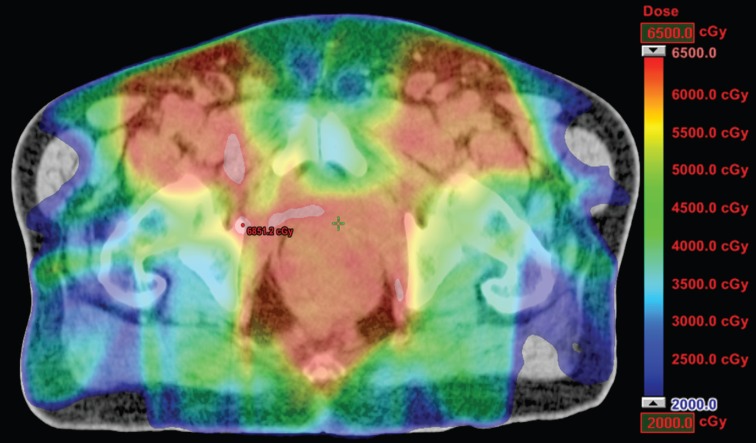
Dosimetric results obtained with IMRT.

**Figure 3.**
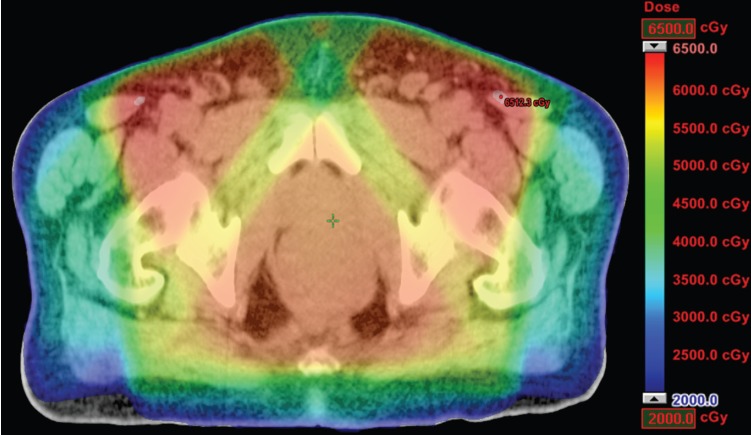
Dosimetric results obtained with conformal radiotherapy.

**Figure 4.**
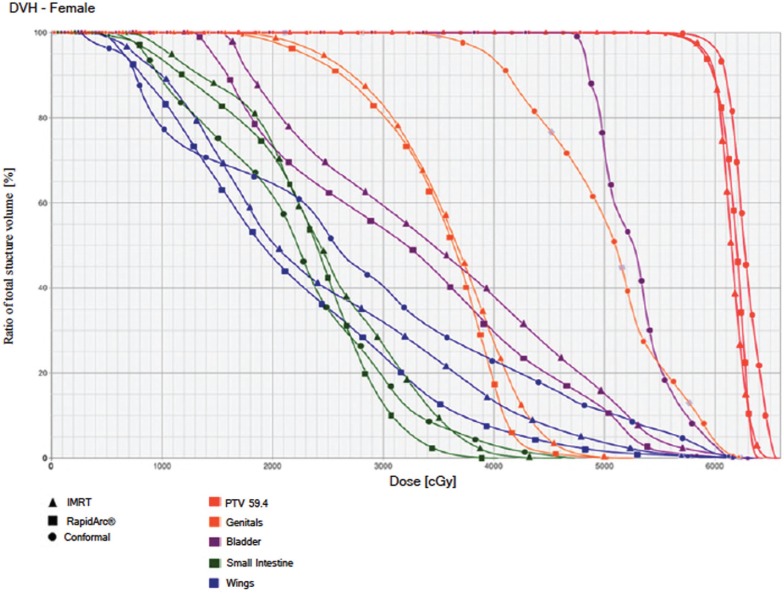
Dose–volume histogram: female.

**Figure 5.**
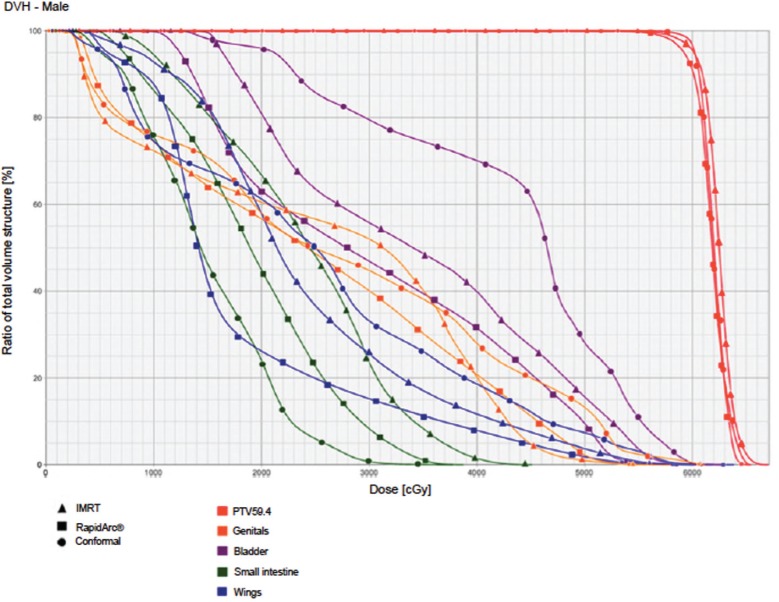
Dose–volume histogram: male.

The IMRT achieved better dosimetric results in all organs tested in both the male and female patients. Compared with conformal radiotherapy, IMRT dose was reduced by 59% in the bladder, 14% in the intestine, 41% in the genitals, 26% in the iliac wings, 75% in the femoral heads and 14% in healthy tissue. The integral doses for all organs were lower with RapidArc® than with the sliding window IMRT or the conformal radiotherapy (Table 3, Figures 1–5).

## Discussion

This study proves the dosimetric benefits of two IMRT techniques compared to the conformal radiotherapy in patients with locally advanced anal canal cancer with compromised bilateral inguinal nodes under similar coverage conditions, which permits valid comparisons regarding homogeneity tumour conformity indexes and dosimetry of organs at risk. The results obtained in the local context replicate the findings of other dosimetric studies that have employed IMRT for anal canal cancer in a different clinical setting since, in this study, patients with locally advanced tumours with compromised bilateral inguinal nodes have been included and have been treated with simultaneous integrated boost.

The toxicity reported in patients with anal canal cancer treated with conformal radiotherapy in association with Mitomycin-C and 5-Fluorouracil is high, particularly regarding haematological, dermatological, and gastrointestinal toxicity. Current reports indicate acute haematologic toxicity of grade 3 or 4 in up to 61% of patients, and non-haematologic acute toxicity of grade 3 or 4 in up to 74%. This toxicity remains as chronic toxicity of grade 3 or 4 at the intestinal, vesical, or skin level in up to 11% of patients [4].

The use of bone-marrow-sparing IMRT in patients with anal canal cancer treated with concomitant chemoradiotherapy demonstrated a decrease in severe haematologic toxicity with figures below 5–10% when average doses below 22.5–25 Gy, respectively, are achieved in the pelvis bone [11]. Another study concluded that the volume of the bony pelvis receiving 10–20 Gy is a good tracer of haematologic toxicity, and therefore, techniques aimed at reducing radiation doses in the bony pelvis should be designed [12]. In fact, a clinical trial being developed is aimed at establishing the benefit of the bone marrow-sparing IMRT in patients with pelvic tumours after this technique demonstrated benefits in a preliminary study [13]. In this report, we have demonstrated that the use of IMRT reduced doses of the iliac wings up to 26%, which meant going from an average dose of 27 Gy with conformal radiotherapy to 18 Gy with IMRT, which is reflected in lower haematological toxicity.

The IMRT not only achieved a reduction in radiation dose of the bony pelvis, but also demonstrated a reduction of up to 59% in the bladder, 14% in the intestine, and 41% in the genitals. This should impact the most common and most severe toxicity events reported in patients with anal canal cancer treated with concomitant chemoradiotherapy. Some preliminary reports have shown a better toxicity profile among patients treated with IMRT [14, 15]; however, no studies have yet been conducted to demonstrate these benefits in the context of a randomized clinical trial.

The potential benefits of the IMRT are not limited to a decrease in toxicity, but would also result in improved adherence to radiotherapy and chemotherapy treatment. Currently, 9% of patients treated in clinical trials do not complete radiotherapy treatment and 5% do not complete chemotherapy [4]. These figures could be higher in routine practice outside of a clinical trial. Nonadherence may affect the effectiveness of the treatment if a phenomenon of tumour repopulation could occur during the interruptions.

Although the two IMRT techniques showed similar dosimetric benefits in organs at risk, RapidArc® reduced the execution time in 75% with respect to the sliding window IMRT. A treatment executed in a quicker manner reduces uncertainties due to error by intrafraction motion, is more comfortable for the patient and allows for a better circulation of patients in radiotherapy services that are high in demand. A potential disadvantage of prolonged execution times observed in sliding window IMRT is a possible decrease in the effectiveness of treatment [16]. RapidArc® also showed a reduction of 77% in the monitor units compared to the sliding window IMRT. A smaller number of monitor units is associated with a decrease, at least theoretical, in the risk of radiation-induced tumours secondary to scattered radiation.

A known disadvantage of RapidArc® compared with the sliding window IMRT or conformal radiotherapy is the integral dose that the healthy tissues receive; however, in this situation, due to the treatment volume, the integral doses received by the healthy organs were lower with RapidArc®. However, the integral doses do not describe the potential risk of radiation-induced tumours that may arise in areas that receive low doses, which are higher when IMRT techniques are employed [17].

The study has limitations since it only includes two patients; however, we consider that the results can be replicated in other patients with locally advanced anal canal cancer considering that the differences observed between the different techniques are important and confirm what has already been observed in other studies. Implementing this technique in a routine manner requires specific training by the radiation oncologist, the medical physicist and the technologist; this technique should always be accompanied by a customized immobilization device and a specific IGRT protocol since the high beam shaping obtained with the IMRT with no IGRT may increase the risk of geographical errors [18]. It is recommended that patients with anal canal cancer be referred to reference centres at the national level with expertise in IMRT and IGRT so that they can benefit from the new technologies available and can be included in studies describing the clinical benefits derived from a better dosimetry.

## Conclusion

IMRT demonstrated having coverage and conformal indexes similar to those of the conformal radiotherapy, with better disometric results in organs at risk which translates into less dermatological, gastrointestinal, and haematologic toxicity with a consequent decrease in treatment interruptions or withdrawals. The IMRT with RapidArc® demonstrated advantages with respect to the sliding window IMRT with regard to the homogeneity of the dose, the treatment execution time, and the administered monitor units. The use of RapidArc® translates into more comfortable treatment for the patient with less uncertainty regarding intrafraction motion and the theoretical potential of a decrease in radiation-induced tumours.

## Figures and Tables

**Table 1.** Dosimetric objectives for inverse planning.

Structure	Dose (Gy)	Volume (%)
PTV 59.4	59.4	≥95
	63.5	≤2
PTV 49.5	49.5	≥95
Bladder	30	≤80
	40	≤40
	50	≤5
Small intestine	30	≤40
	40	≤30
	50	≤5
Genitals	30	≤40
	40	≤20
	50	≤5
Iliac crests	10	≤40
	20	≤30
	50	≤5
Femoral heads	45	≤5

**Table 2.** Dosimetric results for high and low risk PTV.

Dosimetric index	PTV 49.5			PTV 59.4		
	**RapidArc**	**IMRT**	**Conventional**	**RapidArc**	**IMRT**	**Conventional**
**Male**						
V90%	100.0%	99.0%	99.8%	99.9%	100.0%	100.0%
V95%	99.0%	99.0%	97.9%	99.0%	99.0%	100.0%
V107%	58.0%	85.0%	70.3%	7.0%	19.0%	9.8%
D2% (cGy)	6372	6445	6387	6400	6514	6447
D50% (cGy)	5408	5696	5878	6185	6249	6190
D95% (cGy)	5032	5058	4383	5918	6015	6008
D98% (cGy)	4951	4866	4263	5759	5871	5951
Prescription (cGy)	4950	4950	4500	5940	5940	5940
Coverage index	1.00	0.98	0.95	0.97	0.99	1.00
Homogeneity index	1.29	1.30	1.42	1.08	1.10	1.09
Tumor conformity index	0.98	0.97	0.92	0.94	0.97	0.98
Monitor units	827	3902	216	827	3902	226
Treatment time (min)	3:18	2:45	2:28	3:18	2:45	2:24
**Female**						
V90%	100.0%	100.0%	100.0%	100.0%	100.0%	100.0%
V95%	99.0%	99.0%	99.0%	99.0%	99.0%	99.0%
V107%	69.0%	74.0%	89.0%	1.2%	4.0%	29.0%
D2% (cGy)	6336	6369	6502	6347	6399	6521
D50% (cGy)	5959	5984	6119	6194	6149	6264
D95% (cGy)	5004	4968	4593	5903	5919	6031
D98% (cGy)	4899	4860	4457	5782	5790	5929
Prescription (cGy)	4950	4950	4500	5940	5940	5940
Coverage index	0.99	0.98	0.99	0.97	0.97	1.00
Homogeneity index	1.28	1.29	1.44	1.07	1.08	1.10
Tumor conformity index	0.97	0.96	0.97	0.93	0.94	0.98
Monitor units	713	2673	219	713	2673	216
Treatment time (min)	3:03	10:50	2:43	3:03	10:50	2:43

**Table 3.** Dosimetric results for organs at risk.

Structure	Dose (Gy)	Male			Female		
		RapidArc	IMRT	Conventional	RapidArc	IMRT	Conventional
Bladder	Integral dose (Gy * L)	19.5	22.5	28.1	15.2	16.5	25.1
	V30Gy	47%	56%	80%	54%	59%	100%
	V40Gy	31%	40%	70%	30%	37%	100%
	V50Gy	9%	15%	28%	11%	15%	71%
Small intestine	Integral dose (Gy * L)	9.3	11.6	7.3	13.7	14.7	13.2
	V30Gy	8%	23%	1%	12%	26%	19%
	V40Gy	0%	1%	0%	0%	1%	3%
	V50Gy	0%	0%	0%	0%	0%	1%
Genitals	Integral dose (Gy * L)	10.6	11.1	11.6	4.4	4.6	6.4
	V20Gy	57%	60%	58%	97%	99%	100%
	V30Gy	40%	52%	45%	80%	83%	100%
	V40Gy	21%	20%	28%	17%	26%	93%
	V50Gy	2%	1%	13%	0%	0%	56%
Iliac wings	Integral dose (Gy * L)	7.7	10.2	10.5	7.1	7.9	8.9
	V10Gy	87%	93%	74%	84%	89%	77%
	V20Gy	26%	58%	61%	47%	50%	65%
	V30Gy	15%	25%	33%	24%	32%	40%
	V40Gy	8%	11%	18%	7%	13%	23%
	V50Gy	2%	4%	7%	2%	4%	11%
Right femoral head	Integral dose (Gy * L)	5.2	6.1	7.5	4.5	4.5	5.9
	V45Gy	21%	30%	73%	32%	33%	87%
	V54Gy	5%	8%	52%	7%	4%	69%
Left femoral head	Integral dose (Gy * L)	5.2	6.1	7.5	4.5	4.5	5.9
	V45Gy	16%	21%	73%	43%	41%	97%
	V54Gy	5%	4%	56%	14%	9%	90%
Skin	Integral dose (Gy * L)	9.2	10.8	10.8	10.9	11.7	12.4
	Minimum dose (cGy)	0	0	0	76	76	21
	Maximum dose (cGy)	4111	4917	5041	5609	5714	5628
Healtdy tissue	Integral dose (Gy * L)	321.7	372.3	395.2	313.6	334.6	382.9
	V5Gy	78%	80%	77%	77%	75%	74%
	V10Gy	61%	67%	63%	63%	63%	61%
	V20Gy	36%	47%	50%	43%	48%	51%
	V30Gy	23%	30%	33%	26%	32%	37%
